# Coecal volvulus: An acute complication of pregnancy

**DOI:** 10.4103/0974-2700.70771

**Published:** 2010

**Authors:** Youssef Narjis, Khalid Rabbani, Sanaa Largab, Abderaouf Soumani, Benacer Finech, Abdelhamid El Idrissi Dafali

**Affiliations:** Department of General Surgery, Universitary Hospital Mohamed VI, Cadi Ayyad University, Marrakesh, Morocco; 1 Department of Gynecology A, Universitary Hospital Mohamed VI, Cadi Ayyad University, Marrakesh, Morocco

Sir,

Volvulus of the coecum is an extremely rare cause of intestinal obstruction during pregnancy. Indeed, few observations have been published in the literature on this association.[1] Its diagnosis remains difficult, often posed at laparotomy, and its maternal and fetal prognosis is mainly conditioned by early diagnosis and treatment.[1,2]

**Figure 1:**
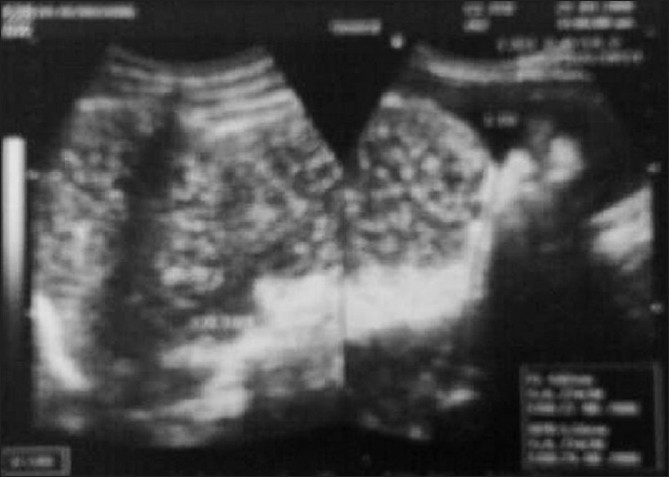
Ultrasonography of our patient, showing peritoneal fluid

A young pregnant woman, 19 years old, nulliparous, primigravidae, was admitted to the emergency surgery with complaints of pain at the right flank, bilious vomiting and distension of the abdomen with no passage of flatus and stools for 4 days. At examination, she had tachycardia and abdominal distension with normal fetal heart sounds. The rectum was empty on rectal examination. At vaginal examination, cervix was closed. Abdominal ultrasonographic examination revealed a monofetal pregnancy of 26 weeks and abundant peritoneal fluid suggesting peritonitis [Figure 1].

**Figure 2:**
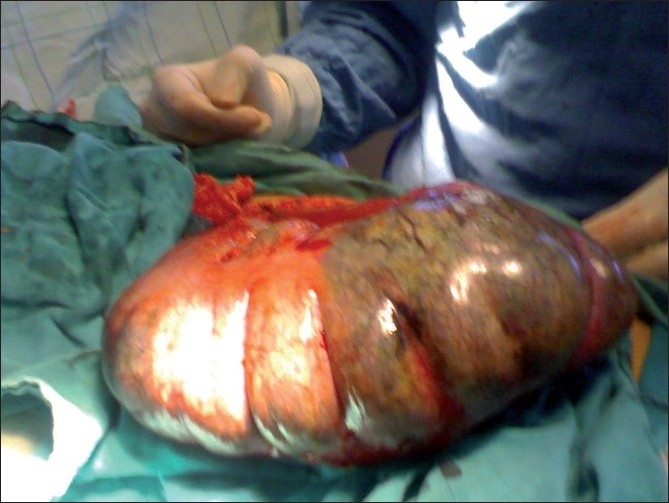
Per operative views showing the necrotic coecum with no fixation

**Figure 3:**
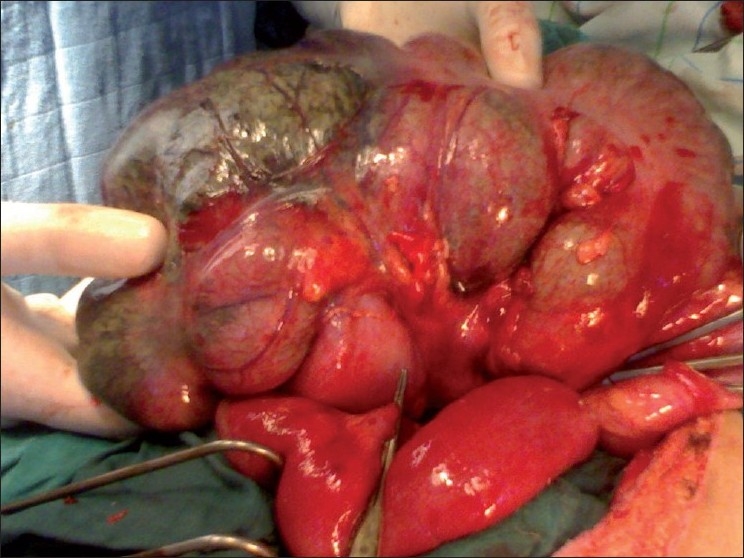
Per operative views showing the necrotic coecum with no fixation

A tocolysis with parenteral beta agonists was started and the patient taken for emergency surgery. The surgical exploration found a necrotic volvulus of coecum and purulent peritoneal fluid confirming peritonitis. There was no peritoneal fixation on the coecum [Figures 2 and 3]. The gangrenous gut was resected and ileocolostomy done. The postoperative course was uneventful. During the restoration of digestive continuity conducted 2 months after the first intervention, the patient presented an exteriorized digestive fistula of the right flank. It dried up spontaneously after 3 weeks. The patient delivered a healthy infant weighing 3.1 kg at full term. The postpartum was uneventful.

Volvulus of the coecum is an axial twist or a folding of the bowel upon its mesentery. This results in acute intestinal obstruction which may or may not be complicated by occlusion of the mesenteric vessels. The condition was first described by Rokitansky in 1837.[1]

Volvulus of the coecum is the second cause of colonic obstruction during pregnancy after sigmoid colon volvulus and its incidence in pregnancy is 1/2500–1/3500.[2] The incidence of coecal volvulus increases with the duration of gestation and is greatest at times of rapid uterine size changes, especially from 16 to 20 weeks, when the uterus becomes an intra-abdominal organ; from 32 to 36 weeks, as the fetus enters the pelvis; and in the puerperium, when the uterine size changes rapidly again.[3,4] In our case, the patient was at the 26^th^ week and cannot be included in these patients.

The diagnosis of this condition is often delayed because the signs and symptoms of intestinal obstruction are mistaken for hyperemesis, placental abruption or ruptured uterus, and also because physicians avoid radiological study due to fears about fetal safety.[4] Our case confirms it because our patient was operated only 4 days after the beginning of symptoms.

The diagnosis of coecal volvulus can be made with abdominal plain X-ray (95% sensitivity). A characteristic coffee-bean deformity may be seen directed toward the left upper quadrant.[5] Abdominal ultrasonography is noninvasive and widely available, and is also applicable in coecal volvulus. The “whirlpool sign” consists of the superior mesentery artery (SMA) wrapped by coils of superior mesentery vein (SMV) and bowel, and can be valuable in diagnosing midgut malrotation and volvulus.[6] In our case, ultrasonography revealed peritoneal fluid. Then, our patient was operated for suspicion of peritonitis. Contrast studies and colonoscopy are not favored for the diagnosis of coecal volvulus.[7]

Treatment of coecal volvulus requires urgent laparotomy in most cases. Colonoscopic nonoperative treatment is possible; but because of high failure rate, it is not recommended. Surgical treatment of coecal volvulus consists of untwisting the bowel, decompressing the distended segments, removing devitalized tissue and preventing recurrence.[8] The surgical techniques described for coecal volvulus are coecostomy, coecopexy, resection with ileostomy and resection with primary anastomosis.[4,9] Laparoscopic coecopexy for primary intermittent coecal volvulus may be an alternative to laparotomy in selected cases but is not indicated in the emergency situation.[9]

The coecopexy technique has low rates of complication and recurrence rates of 0–8%.[4,10] Surgical resection eliminates the possibility of recurrence and usually results in low morbidity and mortality. As pregnancy results in marked displacement of the coecum, which may predispose to relapse of the volvulus, resection is the best procedure to avoid recurrence.[4,11] In our patient, the coecum was necrotic, so we had to do a resection of the coecum. We could not perform anastomosis because we found purulent peritoneal fluid.

We conclude that coecal volvulus is an uncommon cause of colonic obstruction in pregnancy. It is an emergency situation and diagnosis should be withheld quickly by physical examination and usually radiological investigations. Any delay in diagnosis may cause often maternal and fetal complications. The treatment is always surgical.
